# Seasonality and the female happiness paradox

**DOI:** 10.1007/s11135-023-01628-5

**Published:** 2023-02-21

**Authors:** David G. Blanchflower, Alex Bryson

**Affiliations:** 1 Bruce V. Rauner Professor of Economics, Department of Economics, Dartmouth College, NH07355 Hanover, USA; 2 Professor of Quantitative Social Science, Social Research Institute, University College London, London, UK

**Keywords:** Happiness, Unhappiness, Anxiety, Life satisfaction, COVID, Gender, J16, I31

## Abstract

Most studies tracking wellbeing do not collect data across all the months in a year. This leads to error in estimating gender differences in wellbeing for three reasons. First, there are seasonal patterns in wellbeing (particularly life satisfaction and happiness) which are gendered, so failure to account for those confounds estimates of gender differences over time. Second, studies fielded in discrete parts of the year cannot extrapolate to gender differences in other parts of the year. Making inferences about trends over time is particularly problematic when a survey changes its field survey dates across years. Third, without monthly data, surveys miss big shifts in wellbeing that occur for short periods. This is a problem because women’s wellbeing is more variable over short periods of time than men’s wellbeing. It also bounces back faster. We show that simply splitting the data by months in a happiness equation generates a positive male coefficient in one subset of months from September to January and a negative coefficient in months February to August. Such a split has no impact on the male coefficients in an anxiety equation. Months matter.

## Introduction

A large literature has explored gender differences in subjective wellbeing. The findings have resulted in what has been referred to as the female happiness paradox due to what appear to be contradictory results regarding the association between gender and wellbeing depending on the wellbeing metric used. The evidence on unhappiness data is clear cut: always and everywhere no matter what data file or measure is used, which country or time-period is examined, women have poorer mental health than men. In a recent paper Blanchflower and Bryson (2022a) found that is true for a variety of measures including the number of bad mental health days, anxiety, depression, fearfulness, sadness, loneliness, anger and more restless sleep. There is also a good deal of evidence from all sorts of measures of positive affect that women are less satisfied with many aspects of their lives such as democracy, the economy, the state of education and health services. They are also less happy in the moment in terms of peace and calm, enjoyment, cheerfulness, feeling active, vigorous, fresh, rested and saying life is good for me. And yet, across a range of studies, there is also evidence that, in terms of overall life satisfaction and happiness, women score more highly than men. Hence the paradox with women appearing both more and less ‘happy’ than men, depending on the wellbeing metric used.

In the United States Blanchflower and Oswald (2004) found that women were happier than men in both the United States and the United Kingdom. Many subsequent studies have replicated that finding using happiness and life satisfaction data.^1^ At the same time there has been evidence from unhappiness data, for example on distress and despair, bad mental health days, depression and other measures, that women are more unhappy than men (Beja 2018; Blanchflower and Bryson 2022b; Blanchflower and Feir 2022; Blanchflower and Oswald 2020).

In this paper we focus on a major methodological problem which has confounded analysts' estimates of gender differences in wellbeing. Most studies do not collect data across all the months in a year. This is a problem for three reasons. First, there are seasonal patterns in wellbeing (particularly life satisfaction and happiness) which are gendered, so failure to account for those will confound estimates of gender differences in wellbeing over time. Second, if some studies are fielded in discrete parts of the year, this means one cannot extrapolate to gender differences in other parts of the year. Making inferences about trends over time is particularly problematic when a survey changes its field survey dates across years. Third, without monthly data, surveys miss big shifts in wellbeing that occur for short periods. This is a problem because, as we show below, women’s wellbeing is more variable over short periods of time than men’s wellbeing. It also bounces back faster.

We show for the first time that there are significant variations by month regarding whether males are happier than females. For example, in the UK women are less happy than men in January, September, October and November, and happier in March. In the remaining eight months there are no gender differences. On the other hand, splitting by month has no impact on the male coefficients in an anxiety equation for the UK: men are less anxious than women throughout the year. So, the anxiety estimates would show men as less anxious than women whenever a survey is conducted whereas the association between gender and happiness depends on the time of year. If one happened to sample men and women in the UK in Spring and Summer months it would seem that women were happier than men, yet they are more anxious – the paradox.

In addition, women’s happiness varies more than men’s with sudden shocks. Monthly data reveal that women’s happiness was more adversely affected by the COVID shock than men’s, but also that women’s happiness rebounded more quickly suggesting resilience. In contrast, we find little monthly variation in the gender gap in unhappiness. For example, women are consistently more anxious than men, and whilst the gap in anxiety increased during COVID, it varied little month by month.

It is also important for analysts – and policy makers – to be aware of the time dimension associated with wellbeing questions because metrics often refer to different time frames and may be capturing quite different components of wellbeing. For instance, if respondents are asked to reflect on “how life has turned out so far” they may rate their wellbeing quite differently to a scenario in which they are asked about how they were feeling “yesterday”.

We conduct a detailed analysis for the UK with a large data file – the Annual Population Survey, 2012–2021 – that has data on, happiness, life satisfaction and anxiety. Gender differences in these three variables differed a lot pre- and post- the COVID shock. In the years from 2012 to 2017 men’s happiness was above that of women’s, in 2018 they were identical, but from 2019 men had greater happiness than women. The emergence of higher happiness among men is confirmed in regression analyses and is apparent controlling for month of survey. We confirm this lower happiness among women during COVID with data from the new Opinions and Lifestyle Surveys of 2020–2022. A very similar increase in men’s life satisfaction, relative to women’s also occurs from 2018 – so prior to the pandemic – and is robust to month controls. In contrast, women are permanently more anxious than men over time, a difference that seems to have been exacerbated by the pandemic.

We also confirm the importance of having monthly data from an examination of life satisfaction data for the United States from the Behavioral Risk Factor Surveillance System (BRFSS) from 2005–2011 and 2019–2021. We find men’s life satisfaction is higher than women’s between 2005 and 2018 and that it tends to be higher than women’s in four of the twelve months of the year and is never significantly below that of women. Analogously in every month in the period 1993–2021 males are always significantly less unhappy than females when asked about the number of bad mental health days they have experienced in the last month.

The remainder of the paper is structured as follows. Section Two reviews the prior literature. Section Three describes our data and the methodology we use to identify seasonality in wellbeing equations. Section Four presents our results and Section Five discusses them and concludes.

## Prior literature

It is well-known that there is temporal variance in wellbeing. Time use data reveal variance across the course of the day. This is often due to change over the course of the day in the activities individuals are performing and the people they are with (Kahneman et al 2004; Bryson and MacKerron 2017). However, it can also reflect Circadian rhythms which are biochemically regulated processes that generate a diurnal variation in the body’s level of alertness. These are known to affect a range of human behaviours and activities including injury rates among shift workers (Fortson 2004) and student performance in the classroom (Kirby and Kirby 2006). There is also substantial variation in wellbeing over the course of a week which, some maintain, may explain why Monday is the peak day for suicides (Maldonado and Kraus 1991).^2^

Wellbeing also varies with the seasons (Foster and Kreitzman 2010). Harmatz et al (2000) found depression, hostility, anger and irritability are highest in winter and lowest in summer with females having stronger seasonal variation in wellbeing than males. Some link seasonal variance on wellbeing, to variance in daylight hours and weather conditions, although wellbeing responses to such external factors are heterogeneous (Leibowitz and Vitterso 2020; Winthorst et al. 2020).

There is also a vast literature tracking changes in individuals’ wellbeing when they experience particular events, such as marriage, divorce, bereavement, and unemployment. These studies tend to find that such events lead to shifts in wellbeing but, notwithstanding methodological problems with the literature, there is often a return to pre-existing levels of wellbeing – at least to some large degree – after a period. This process, which is akin to mean reversion, is often termed ‘adaptation’ (Luhmann et al. 2012).

Some events are experienced as negative shocks which can have sudden adverse consequences for individuals’ wellbeing. But even here, there is evidence of mean reversion. For instance, Bryson and MacKerron (2022) show proximity to terrorist-related events such as bombings and shootings, negatively impacts individuals’ momentary wellbeing if the event involved death, but the effects disappear over a 72-h period.

Similar findings are apparent in other investigations into the impact of terrorist-related events on wellbeing (Schlenger et al. 2002; Schuster et al. 2001; Silver et al. 2002; Metcalfe et al. 2011; Clark et al. 2020; Krueger 2007). Kimball et al. (2006) examined high frequency weekly data for the US on happiness from the Monthly Surveys of Consumers in August, September and October 2005 at the time when Hurricane Katrina hit New Orleans. The levy was breached on August 29th, 2005. The authors found that happiness dipped significantly in the US during the first week of September after the seriousness of the damage became clear. The dip in happiness lasted two or three weeks in South Central but returned to normal in the rest of the country in one to two weeks. Interestingly, men did not have significantly different responses to Katrina than women. They also found some evidence that happiness dipped briefly in the week of October 13–19 after news of an earthquake hitting Pakistan and India on October 8, 2005.

Such findings are consistent with an established psychological literature which suggests individuals have subjective wellbeing “set points” (for example, Cummins and Wooden 2014), to which they are anchored over the long run. When faced with unfortunate events people deviate from these set points, only to return to them not long afterwards.

The literature on shocks and wellbeing has come to the fore recently with the onset of the COVID pandemic. In earlier work tracking mental health in the United States using the US Census Bureau’s Household Pulse Survey we showed that, in the period through to December 2021, men had lower levels of anxiety, worried less and were less likely than women to say they were unhappy and depressed in 2020 and 2021 (Blanchflower and Bryson 2022b). Proto and Quintana-Domeque (2021), Pierce et al. (2021) and Banks et al. (2021) also found increases in mental distress in the UK, measured with the 12-item General Health Questionnaire (GHQ12) scores in the UK Household Longitudinal Survey (UKHLS), that were greater among women than they were among men. Although these gender differentials are apparent pre-pandemic in other studies, some maintain that COVID may have exacerbated them due to the uneven distribution of household chores and childcaring responsibilities brought on, in part, by the pandemic (Giurge et al. 2021).

The COVID pandemic also negatively impacted individuals’ mental wellbeing through lockdowns intended to limit the spread of the virus. Using data for the United States, Adams-Prassl et al. (2022) find women have poorer mental health than men during COVID, and that this effect was exacerbated by COVID induced lockdowns. Indeed, the ‘lockdown’ effect was confined to women.

The persistence of negative affect among women during COVID is consistent with the proposition that women take more time than men to revert to their previous wellbeing levels when faced with a shock. Becchetti and Conzo (2021) argue they find support for this proposition based on a regression they ran on a variable which asked, in 2006 and 2012 in the ESS:

### *Q1.*

*"When things go wrong in my life it takes a long time to get back to normal?”*

The distribution of responses on the ordinal agree/disagree scale is:

**Table:** 

	Men	Women
Disagree strongly	11	8
Disagree	42	38
Neither	23	25
Agree	19	24
Agree strongly	4	6
N	45562	54426

We replicated their finding that males are significantly less likely to agree to the statement.^3^ However, as we show later, we find big swings in positive affect (life satisfaction and happiness) with the onset of COVID among both men and women, with women in particular taking a bigger wellbeing hit, only to recover rapidly. As background Goldin (2022), noted “*compared with previous recessions, the one induced by COVID-19 impacted women’s employment and labor force participation somewhat more relative to men’s and thus deserves the moniker “she-cession*”.

## Data and methodology

We analyze seasonality in men’s and women’s wellbeing in the United Kingdom using the *Annual Population Survey* (APS) and in the United States using *the Behavioral Risk Factor Surveillance System* (BRFSS).

Our micro-analyses for the UK use the APS over the period 2012–2021. These data are administered and made available for research by the Office for National Statistics (ONS). The APS combines survey records from two sources. The first source is the *Quarterly Labour Force Survey* (QLFS). The QLFS is a rolling, quarterly panel survey in which respondents are interviewed over five successive quarters before exiting the sample. The APS includes records from waves 1 and 5 of the QLFS across the four quarters of the calendar year in question. The second source is the *Local Labour Force Survey* (LLFS). The LLFS is a rolling annual panel in which respondents are interviewed over four successive years. The LLFS sample is divided into four waves and the APS includes records from all four waves of the LLFS for the calendar year in question.

In addition to the large sample sizes, these data have two important advantages over many other studies. First, the survey is conducted throughout the year, and the month of survey interview is recorded in the data. This allows us to control for seasonal patterns in wellbeing data, which have been identified in the past, especially in suicides.

Second, the APS contains information on three, 11-step, wellbeing variables.^4^ The questions asked are as follows, with respondents answering on a scale of 0 to 10.

### *Q2.*

*"Life satisfaction—Overall, how satisfied are you with your life nowadays?”.*

### *Q3.*

*“Happiness—Overall, how happy did you feel yesterday?”.*

### *Q4.*

*“Anxious—Overall, how anxious did you feel yesterday?".*

ONS reports trends in these wellbeing measures quarterly.^5^ The first of these variables refers to the integral of how life has been going and is unlikely to change quickly as compared with happiness and anxiety which relate directly to what happened the previous day.

Weights are provided by ONS to make the APS dataset representative of the general population in a given year. We run pooled years Ordinary Least Squares (OLS) regressions for each of the wellbeing outcomes above for the period 2012–2021 of the following form:

$$W_{it} = \beta_{0} + \beta_{1} male + \beta_{2} race + \beta \prime_{3} country + \beta \prime_{4} year + \beta \prime_{5} month + \beta \prime_{6} MonthXyear_{{}}$$

where ${W}_{it}$ denotes the wellbeing of individual *i* in a given month and year; $\beta {\prime }_{4}$ are the coefficients for a vector of year dummies; $\beta {\prime }_{5}$ are the coefficients for a vector of month dummies; $\beta {\prime }_{4}$ interacts month and year for recent periods to capture COVID-related movements in wellbeing. Controls are included for race, country of residence and being male. In variants of this model,we incorporate age and age squared. We also run separate models for men and women to establish whether there are differences in men’s and women’s wellbeing across seasons in the year. In addition, we run 118 monthly happiness regressions to examine month-by-month variation in the male coefficient, having controlled for age, age squared, and country of residence.

With the APS data we trace the monthly path of wellbeing since January 2012, including the COVID pandemic which began in March 2020, with weights imposed.^6^ We identify gendered patterns in the seasonal fluctuations which have not been identified before.

We supplement these estimates with trends in wellbeing from the *Opinions and Lifestyle Survey* (OALS) in the UK from March 2020 through to February 2022.^7^ As in the case of the APS, the questions regarding happiness and anxiety refer to 'yesterday'.

### *Q5.*

*“Overall, how happy did you feel yesterday?".*

### *Q6.*

*"Overall, how anxious did you feel yesterday?".*

These questions are answered on a scale of 0 to 10, where 0 is "not at all" and 10 is completely". Again, the ONS publishes regular reports by gender on these two variables.^8^

We run similar regression analyses for wellbeing in the United States using the BRFSS. This is the only other major data file that we are aware of that contains both wellbeing – specifically happiness and the number of bad mental health days – which also has full coverage by month.

The BRFSS is a telephone survey conducted by the Center for Disease Control (CDC) regarding health-related risk behaviors, chronic health conditions, and use of preventive services. Established in 1984 with 15 states, BRFSS now collects data in all 50 states as well as the District of Columbia and three territories of the United States. In recent years around 400,000 adult interviews are conducted each year in the BRFSS. We analyze these data for the period 2005–2018.

The life satisfaction equation was not included in the main survey after 2010 but was included in a subset of states, hence we always include state dummies. The question used is:

### *Q7.*

*In general, how satisfied are you with your life? Very satisfied (= 4), satisfied (= 3), dissatisfied (= 2), very dissatisfied (= 1).*^9^

Sample sizes are over 2.4 million and we present results overall and separately by month. The model takes a similar form to that presented above for the UK, with each equation including a full set of year dummies, race and state of residence along with a sex dummy.

Data is also available on the number of bad mental health days over the prior month, coded 0–30, for the period 1993–2018 on nearly 8 million respondents as well as for the most recent period 2019–2021 for 800,000 respondents. Respondents are asked:

### *Q8.*

*“Now thinking about your mental health, which includes stress, depression, and problems with emotions, for how many days during the past 30 days was your mental health not good?”.*

We adopt a similar modelling strategy as that described above with the APS.

## Empirical evidence for the UK and the USA

### Happiness, Life Satisfaction and Anxiety in the UK

The top half of Table 1 shows annual mean values for the three APS wellbeing measures for women and men separately. Women’s happiness is above that for men from 2012–2017 (columns 1 and 2). In 2018 they were the same but from 2019–2021 the pattern switched, and males were happier than females. In the case of life satisfaction, the switch occurred in 2020 (columns 3 and 4). In contrast in every year women were more anxious than men (columns 5 and 6). The paradox is apparent in early years but there are indications of men being happier and more satisfied than women in recent years. A good deal seems to have to do with COVID in the period since March 2020, but the trend had started before then for happiness.

**Table 1:** Annual mean wellbeing by year and month by gender, APS 2012–2021

	Happy		Life satisfaction		Anxious	
	Male	Female	Male	Female	Male	Female
2012	7.28	7.34	7.4	7.48	2.93	3.15
2013	7.35	7.4	7.45	7.52	2.83	3.08
2014	7.43	7.46	7.55	7.6	2.75	3.04
2015	7.48	7.5	7.63	7.67	2.7	3.02
2016	7.49	7.52	7.65	7.69	2.72	3.05
2017	7.53	7.55	7.67	7.7	2.72	3.07
2018	7.55	7.55	7.7	7.71	2.64	3.03
2019	7.55	7.54	7.68	7.7	2.72	3.12
2020	7.39	7.3	7.52	7.46	3.05	3.58
2021	7.47	7.39	7.49	7.41	2.84	3.35
January	*7.35*	7.31	7.53	7.54	2.83	3.23
February	7.41	7.41	7.55	7.58	2.81	3.13
March	7.37	7.42	7.52	7.59	2.87	3.18
April	7.48	7.48	7.59	7.6	2.76	3.12
May	7.48	7.51	7.59	7.61	2.78	3.13
June	7.51	7.53	7.6	7.62	2.75	3.1
July	7.54	7.58	7.61	7.63	2.71	3.02
August	7.53	7.54	7.61	7.64	2.73	3.08
September	*7.47*	7.44	7.57	7.6	2.77	3.19
October	*7.43*	7.4	7.58	7.58	2.82	3.19
November	7.39	7.39	7.58	7.59	2.84	3.22
December	*7.46*	7.45	7.59	7.59	2.73	3.13

Source: UK annual population surveys

The bottom half of Table 1 reports mean rates of happiness, life satisfaction and anxiety by month. In every month anxiety is higher for women than men. For happiness men have higher scores in September, October, December and January and they are the same in December, and lower in all the other months. In the case of life satisfaction, the scores for men are lower than those for women from January to September and the same in the final three months of the year.

**Fig. 1:**
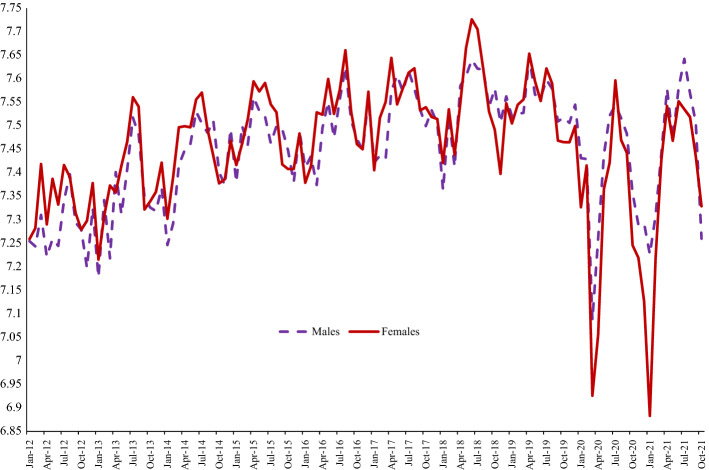
Monthly Happiness by Gender, UK, APS 2012–2021

Figure 1 indicates that women’s happiness “yesterday” was above that of men’s until 2019, after which men experienced greater happiness than women. Both women and men experienced a huge drop in their happiness with the onset of the COVID pandemic, but the fall began nearly a year earlier from April 2019. Perhaps equally remarkable is the recovery in happiness shortly afterwards, with women’s happiness reaching its pre-pandemic levels again as soon as July 2020. However, this was followed by a second plunge in happiness for both sexes by early 2021, a drop which was much more severe for women – their happiness rating was 0.35 points lower than men’s in January 2021. Having risen once again to pre-pandemic levels in June 2021 again, it began to fall as we moved into autumn. The picture is one of great volatility in the momentary happiness of men and women, though that volatility appears greater for women. These sharp reversals, both during COVID and prior to COVID, would not have been observed without high frequency data.

Next, we turn to gender differences over time in life satisfaction. Before coming to the monthly data in the APS it is worth reflecting on the size of the drop in life satisfaction which occurred with the onset of COVID. We can observe this is the long time-series available through Eurobarometer which has included a 4-step life satisfaction question since the 1970s which is:

#### *Q9.*

*“On the whole, are you very satisfied, fairly satisfied, not very satisfied or not at all satisfied with the life you lead? Not at all satisfied (= 1); not very satisfied (= 2); fairly satisfied (= 3) and very satisfied (= 4)”.*

The trends in these scores are plotted in Appendix Figure. 10 for the UK for men and women using the 2003–2021 Eurobarometer files and the Mannheim Eurobarometer Trend files of 1973–2002. Life satisfaction rose steadily over time for men and women and female life satisfaction was above the male rate in the majority of years. However, life satisfaction for both plummets with the COVID pandemic. The female life satisfaction score for 2021 is below its prior historic lows while for men it is on a par with lows only previously seen in 1995 and the mid-1970s. Apart from in 2008, and in the most recent data for 2021, female happiness is above male happiness.

Returning to the APS data for the period since 2012 Fig. 2 runs identical descriptive analyses for trends in life satisfaction as we undertook for happiness in Fig. 1. It looks different to the happiness data. First, variance over time tends to be lower than in the case of happiness. Second, there is only one big dip in life satisfaction during the pandemic, not two. But, again, as in the case of happiness, life satisfaction begins to dip prior to the onset of COVID in around Spring of 2019 and, as in the case of happiness, women’s life satisfaction is higher than men’s until late on in the series with the gap opening up during COVID as women’s life satisfaction took a much bigger hit than men did. Although, again, as in the case of happiness, it bounces back quickly only to start dipping once more in Autumn 2021 (Appendix Table 10).

**Fig. 2:**
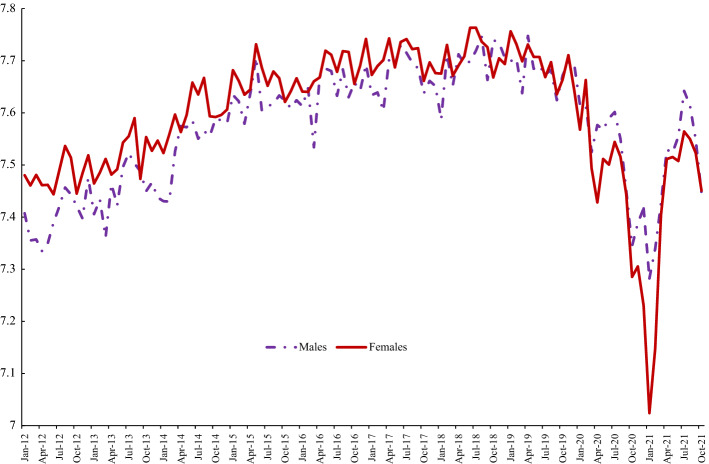
Life Satisfaction by Gender, UK, APS 2012–2021

**Fig. 3:**
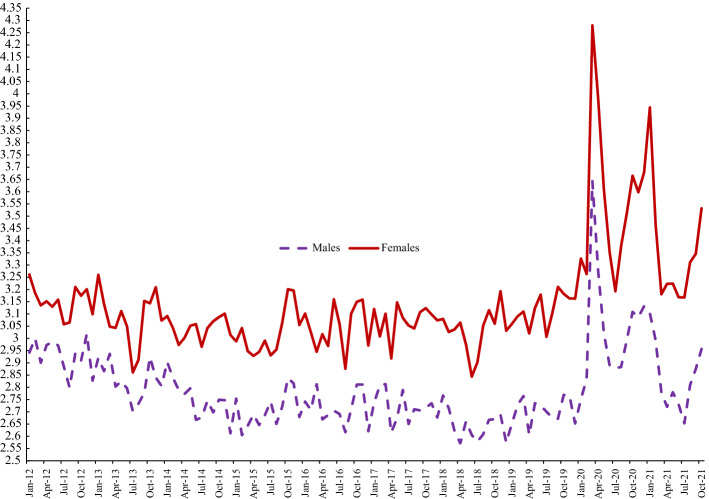
Anxiety by Gender, UK, APS 2012–2021

Figure 3 presents trends in anxiety by gender. Throughout, the female series is always above the male series. As in the case of happiness, we observe two big hits to wellbeing during COVID. Anxiety spikes as COVID hits in March 2020 and again at the beginning of 2021, with both spikes much greater for women than for men. There are signs of another spike in October 2021 too. Throughout the COVID period the gap between women’s anxiety and that of men’s appears to have grown.

**Fig. 4:**
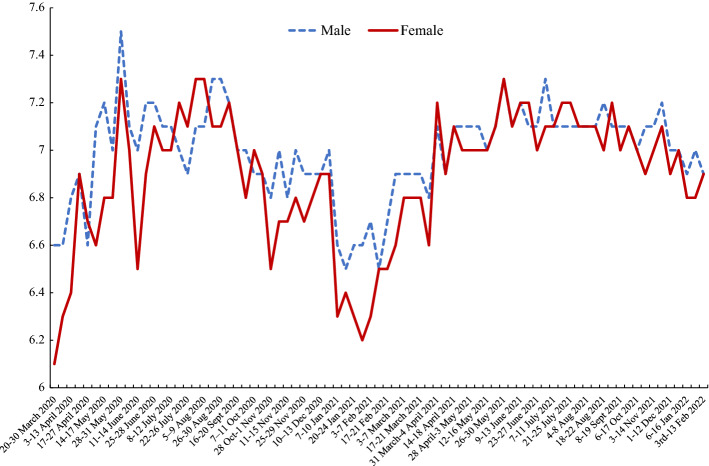
Male and Female Happiness, March 2020-February 2022, UK Opinions and Lifestyle Survey

**Fig. 5:**
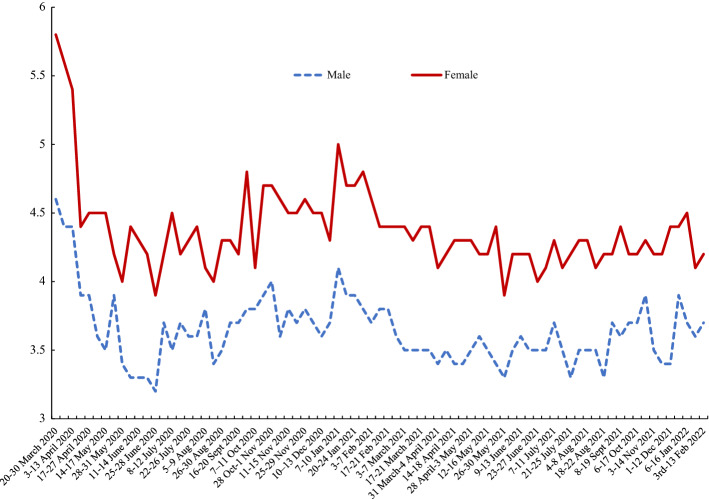
Male and Female Anxiety, March 2020-February 2022. UK Opinions and Lifestyle Survey

Figures 4 and 5 use new data by gender from the *Opinions and Lifestyle Survey* (OALS) in the UK introduced in Section Three. The data run from March 2020 through to February 2022. The time paths for men and women’s happiness in Fig. 4 from the OALS are very similar to those reported in Fig. 1 using the APS data, although the levels in the OALS are lower. Both have lows in March 2020 with recovery to a peak around July 2020 and a major trough at the start of 2021.^10^ The low for females of 6.1 in 20–30 March 2020 is well below that of men of 6.6. The low in January 2021 is also a lot lower for women than men. For example, in the survey for 27–31 January the male happiness score was 6.6 versus 6.2 for females.^11^ The APS data stopped in October 2021, having started falling; the extent of that drop looks greater than in the OALS data. By the start of February 2022 happiness levels were above their March 2020 levels but below what they had been in the summer of 2021 before omicron. Male and female happiness rates were both 6.9.

Figure 5 plots the anxiety rates in OALS. As in the case of the APS female anxiety scores are above male rates, with a differential that is roughly constant over time apart from in March–April 2020 when women’s anxiety is falling more quickly than for men. This corresponds to the dip following the huge spike with the onset of COVID depicted in Fig. 3 in the APS.

Table 2 pools all the years of data together and reports separate OLS estimates for the three wellbeing variables. For the period since January 2020 the model identifies the exact month and year of the survey interview, leaving out March 2020 (the moment COVID arrived) as the reference category. Prior to 2020 we incorporate year and month separately to account for seasonal effects. Column 1 presents the happiness equation. The male main effect is negative and statistically significant, confirming the greater happiness of women in earlier years, but the male interaction effects – all of which are positive and statistically significant – confirm what we observe in the raw data in Fig. 1, namely that women’s happiness started to deteriorate relative to men’s as early as 2018.

**Table 2:** OLS UK wellbeing equations APS 2012–2021

	Happiness	Life satisfaction	Anxious
Male	− 0.0296 (6.76)	− 0.0420 (11.29)	− 0.3100 (52.71)
Male*2021	0.1247 (8.16)	0.1376 (10.61)	− 0.2390 (11.69)
Male*2020	0.1238 (9.14)	0.1203 (10.47)	− 0.2367 (13.06)
Male*2019	0.0460 (3.81)	0.0468 (4.56)	− 0.1298 (8.02)
Male*2018	0.0258 (2.16)	0.0464 (4.57)	− 0.1032 (6.43)
2012	0.2239 (9.22)	− 0.0970 (4.71)	− 0.8470 (26.06)
2013	0.2832 (11.68)	− 0.0532 (2.59)	− 0.9229 (28.43)
2014	0.3539 (14.59)	0.0311 (1.51)	− 0.9676 (29.80)
2015	0.3956 (16.30)	0.1034 (5.02)	− 1.0076 (31.01)
2016	0.4146 (17.03)	0.1238 (5.99)	− 0.9936 (30.49)
2017	0.4520 (18.57)	0.1509 (7.31)	− 0.9935 (30.50)
2018	0.4434 (17.89)	0.1378 (6.56)	− 0.9955 (30.02)
2019	0.4281 (17.28)	0.1278 (6.09)	− 0.9225 (27.83)
Jan-20	0.4102 (13.07)	0.0777 (2.92)	− 0.8746 (20.81)
Feb-20	0.3293 (10.51)	0.0846 (3.18)	− 0.7654 (18.25)
Apr-20	− 0.0040 (0.12)	− 0.0511 (1.74)	− 0.0933 (2.01)
May-20	0.2300 (7.15)	− 0.0321 (1.18)	− 0.4536 (10.54)
Jun-20	0.2502 (7.37)	− 0.0631 (2.19)	− 0.6312 (13.90)
Jul-20	0.3615 (10.95)	− 0.0109 (0.39)	− 0.6763 (15.30)
Aug-20	0.3322 (10.61)	− 0.0353 (1.33)	− 0.6364 (15.19)
Sep-20	0.3505 (10.59)	− 0.1210 (4.31)	− 0.5792 (13.07)
Oct-20	0.2318 (6.57)	− 0.2115 (7.07)	− 0.4565 (9.67)
Nov-20	0.2131 (6.92)	− 0.2014 (7.71)	− 0.5644 (13.69)
Dec-20	0.0817 (2.45)	− 0.2295 (8.10)	− 0.3251 (7.27)
Jan-21	0.0127 (0.39)	− 0.3956 (4.40)	− 0.2983 (6.88)
Feb-21	0.1907 (5.63)	− 0.3283 (1.43)	− 0.6174 (13.62)
Mar-21	0.3136 (9.36)	− 0.1532 (5.39)	− 0.7971 (17.77)
Apr-21	0.4390 (12.74)	− 0.0329 (1.13)	− 0.8622 (18.69)
May-21	0.3420 (10.38)	− 0.0179 (0.64)	− 0.8183 (18.55)
Jun-21	0.4223 (12.20)	− 0.0397 (1.35)	− 0.8897 (19.21)
Jul-21	0.3833 (11.17)	0.0311 (1.07)	− 0.7889 (17.17)
Aug-21	0.3889 (11.95)	0.0132 (0.48)	− 0.7455 (17.12)
Sep-21	0.3773 (11.10)	− 0.0177 (0.61)	− 0.7271 (15.97)
Oct-21	0.2846 (5.37)	− 0.0667 (1.48)	− 0.6889 (9.72)
February	0.0771 (8.13)	0.0243 (3.03)	− 0.0504 (3.97)
March	0.1100 (11.92)	0.0135 (1.73)	− 0.0720 (5.83)
April	0.1393 (14.89)	0.0208 (2.62)	− 0.0867 (6.92)
May	0.1636 (17.40)	0.0404 (5.07)	− 0.0681 (5.41)
June	0.1808 (19.41)	0.0582 (7.38)	− 0.0697 (5.59)
July	0.2000 (21.58)	0.0503 (6.40)	− 0.1299 (10.74)
August	0.1778 (18.98)	0.0582 (7.33)	− 0.0981 (7.83)
September	0.1064 (11.47)	0.0367 (4.67)	− 0.0279 (2.25)
October	0.0790 (8.33)	0.0294 (3.66)	− 0.0069 (0.55)
November	0.0499 (5.35)	0.0324 (4.10)	0.0275 (2.21)
December	0.1275 (13.69)	0.0530 (6.72)	− 0.0802 (6.44)
_cons	7.181	8.1233	3.5011
Adjusted R^2^	0.0078	0.0097	0.0101
N	1,453,690	1,454,283	1,452,481

Excluded January, March 2020; controls included for race and country of residence. Source: Annual Population Surveys

There are strong seasonal effects in happiness: January (the excluded category) is the least happy month, while July is the happiest. We also incorporate monthly dummies for the COVID period, distinguishing 2020 and 2021. Relative to March 2020, which is the beginning of the pandemic, the coefficients for January and February are large, positive and statistically significant. Happiness in April 2020 is indistinguishable from March 2020, but happiness bounces back quickly in May–July 2020 so that, by July 2020 it had returned to its pre-pandemic level. Happiness continues to fluctuate by month, dropping again in January 2021, then rising quickly once again.

Column 2 presents an identical model but for life satisfaction. January is the low point in the year for life satisfaction, as it was for happiness. Although there is significant seasonal variance in life satisfaction it is less pronounced than for happiness. Furthermore, temporal change in life satisfaction during COVID looks different to temporal variance in happiness. As in the case of happiness, life satisfaction drops when COVID hits in March 2020, but the initial effect is weak and is not statistically significant in some of the early post-pandemic months. However, the negative effect is apparent strengthens by September 2020 and remains large through to early 2021. Whereas men had lower life satisfaction than women, the interaction between male and year dummies is positive and statistically significant since 2018.

In column 3, anxiety also shows seasonality, peaking in November and again in January. It is lowest in July. During COVID it peaks in the first month (March 2020), returning to pre-pandemic levels about a year later. The male coefficient is negative and so are the male interaction terms suggesting a bigger negative effect at the end of the period for males in the pandemic.

Table 3 focuses on the monthly changes in 2020 and 2021 for happiness in separate equations for men and women. They contain the same controls as in Table 2. It uses the data for the longer time period 2012–2021. The separate estimates by gender reveal not only that there is substantial monthly variance in wellbeing, but that these monthly patterns differ by gender. January is the least happy month followed by November for both men and women while July is the happiest but there are gender differences in the monthly rank order in happiness in the rest of the year.

**Table 3:** UK happiness equations with month fixed effects, 2012–2021 by gender

	Male		Female	
2012	0.0870 (2.64)	0.0469 (1.37)	0.2513 (8.05)	0.2661 (8.23)
2013	0.1584 (4.81)	0.1195 (3.50)	0.2995 (9.60)	0.3149 (9.75)
2014	0.2284 (6.93)	0.1908 (5.59)	0.3698 (11.85)	0.3849 (11.92)
2015	0.2746 (8.32)	0.2368 (6.93)	0.4088 (13.08)	0.4232 (13.09)
2016	0.2849 (8.62)	0.2475 (7.23)	0.4307 (13.76)	0.4487 (13.84)
2017	0.3229 (9.78)	0.2850 (8.33)	0.4679 (14.96)	0.4857 (14.99)
2018	0.3390 (10.26)	0.2994 (8.74)	0.4640 (14.82)	0.4792 (14.77)
2019	0.3436 (10.39)	0.3048 (8.91)	0.4487 (14.32)	0.4636 (14.31)
Jan-20	0.2744 (6.32)	0.3496 (7.70)	0.3202 (7.74)	0.4575 (10.57)
Feb-20	0.2202 (5.08)	0.2242 (4.93)	0.3563 (8.66)	0.4119 (9.55)
Apr-20	0.0727 (1.50)	0.0186 (0.37)	− 0.0118 (0.26)	− 0.0216 (0.46)
May-20	0.3021 (6.74)	0.2218 (4.74)	0.2698 (6.38)	0.2373 (5.38)
Jun-20	0.3353 (7.05)	0.2399 (4.86)	0.3097 (6.91)	0.2580 (5.54)
Jul-20	0.3644 (7.86)	0.2478 (5.14)	0.5189 (11.96)	0.4497 (9.96)
Aug-20	0.3476 (7.97)	0.2582 (5.66)	0.4404 (10.74)	0.3901 (9.09)
Sep-20	0.3121 (6.72)	0.2685 (5.56)	0.3730 (8.56)	0.4136 (9.12)
Oct-20	0.2028 (4.07)	0.1822 (3.53)	0.1986 (4.28)	0.2702 (5.61)
Nov-20	0.1514 (3.55)	0.1750 (3.91)	0.1535 (3.80)	0.2423 (5.73)
Dec-20	0.1134 (2.45)	0.0510 (1.06)	0.0856 (1.93)	0.1041 (2.25)
Jan-21	0.0223 (0.52)	0.0975 (2.15)	− 0.1938 (4.73)	0.0565 (1.32)
Feb-21	0.1712 (3.75)	0.1752 (3.68)	0.1470 (3.41)	0.2026 (4.51)
Mar-21	0.2850 (6.04)	0.2851 (6.04)	0.3363 (7.58)	0.3363 (7.58)
Apr-21	0.3845 (8.21)	0.3304 (6.78)	0.5343 (12.16)	0.5245 (11.48)
May-21	0.3495 (7.93)	0.2692 (5.84)	0.4327 (10.33)	0.4002 (9.15)
Jun-21	0.4311 (9.14)	0.3357 (6.85)	0.5402 (12.25)	0.4885 (10.65)
Jul-21	0.4471 (9.66)	0.3305 (6.86)	0.4936 (11.23)	0.4244 (9.29)
Aug-21	0.4043 (9.25)	0.3150 (6.89)	0.4976 (12.10)	0.4473 (10.40)
Sep-21	0.3464 (7.50)	0.3029 (6.30)	0.3957 (9.14)	0.4364 (9.69)
Oct-21	0.1592 (2.16)	0.1386 (1.85)	0.3316 (4.65)	0.4033 (5.57)
February		0.0712 (5.17)		0.0816 (6.26)
March		0.0752 (5.61)		0.1372 (10.83)
April		0.1293 (9.48)		0.1471 (11.48)
May		0.1555 (11.37)		0.1698 (13.16)
June		0.1706 (12.60)		0.1889 (14.77)
July		0.1918 (14.22)		0.2065 (16.24)
August		0.1646 0(12.08)		0.1876 (14.61)
September		0.1188 (8.79)		0.0967 (7.61)
October		0.0959 (6.97)		0.0657 (5.05)
November		0.0516 (3.82)		0.0485 (3.78)
December		0.1377 (10.23)		0.1189 (9.26)
_cons	7.4377	7.3645	7.2435	7.1087
Adjusted R^2^	0.0103	0.0109	0.0055	0.0062
N	642,432	642,432	811,258	811,258

Excluded March 2020. Controls include country, age and its square and race: Source: Annual population surveys

The ordering by month is as follows:

- Males–January, November, February, March, October, September, April, December, May, June, August, July.
- Females–January, November, October, February, September, December, March, April, May, August, June, July.

The results for 2020 and 2021 presented here are different from those in other studies because they tend not to have coverage over a year. For example, the Eurobarometer data for 2020 and 2021 used earlier is taken from two surveys in both 2020 and 2021.^12^ The data for 2020 is from Eurobarometer #93.1 and #93.2 drawn in July–August 2020 and August–September 2021; for 2021 Eurobarometers #94.3 (February–March) and #95.1 (March–April) were used. The concern is that these surveys may miss the kind of monthly variations we have identified here. This makes it difficult to track movement in wellbeing over time because any movements may simply reflect seasonal differences in wellbeing which coincide with the time of year the survey was undertaken.

**Fig. 6:**
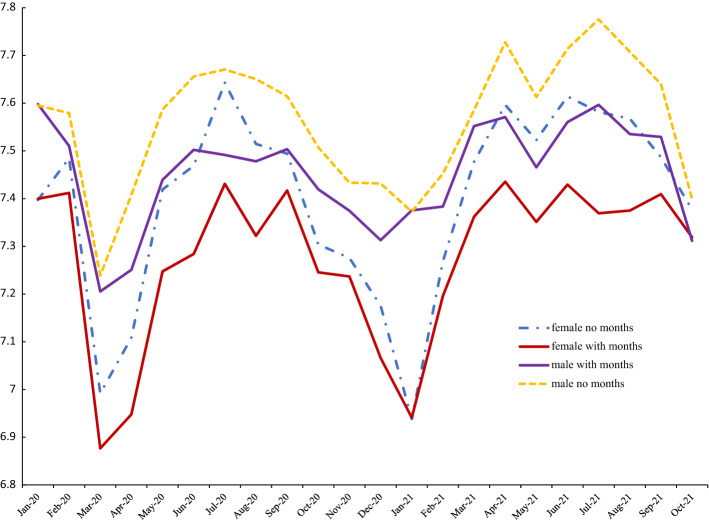
Happiness with and without month effects, 2020–2021

**Fig. 7:**
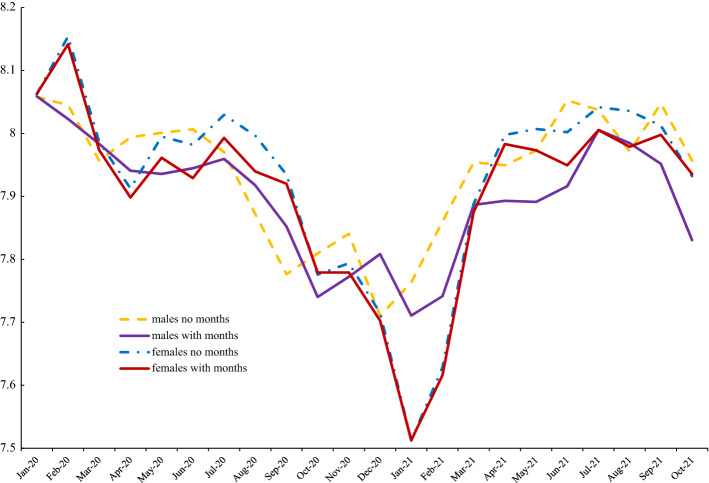
UK Life satisfaction with and without month effects, 2020–2021

**Fig. 8:**
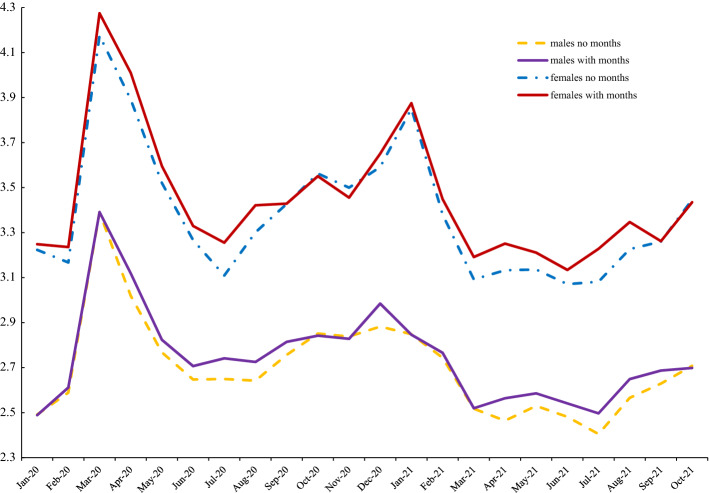
UK anxiety with and without month effects, APS 2020–2021

To illustrate what patterns in men’s and women’s wellbeing looks like with and without month controls we present trends in the APS for the COVID period for happiness, life satisfaction and anxiety respectively in Figs. 6, 7 and 8. From Fig. 6 it is apparent that the line for women with month controls included is well below that of the estimate without seasonal adjustment. Also, with month controls, the female series is below that of men until the last data point in October 2021. Men are happier than women here in 17/18 months. Figure 7 shows smaller differences between seasonally adjusted and unadjusted estimates for life satisfaction. Females saw a much larger dip in life satisfaction at the end of 2020 and the start of 2021 but caught up quickly by March 2021 and for most of the rest of 2021 females had higher levels of life satisfaction. In contrast to happiness and life satisfaction, Fig. 8 shows that seasonal adjustment makes little difference to the gender gap in anxiety: women are always more anxious than men.

Table 4 reports the male coefficients from estimating 118 separate happiness 'yesterday' regressions with the APS for every month from March 2012 to October 2021. Results are only reported if the male coefficient is significantly different from zero, whatever the sign. Sample sizes each month are smaller at the end of the period but average around 12,000 observations. In ninety months, the male coefficient is insignificantly different from zero. In the period through to the middle of 2019 there are eighteen months where the coefficient is significant and negative. But in the ten months from September 2019 the sign switches to *positive* as COVID hits. Of particular note is the positive in September 2019 because it suggests that the move to a positive male coefficient started prior to the pandemic and was exacerbated by it.

**Table 4:** Happiness regressions by month in the APS for the UK–coefficient (*t* value > 1.9) on male dummy

January 2012–October 2021 = 118 monthly regressions		
2012		
March	− 0.0932 (2.38)	13,111
May	− 0.0840 (2.12)	13,209
July	− 0.0676 (1.93)	16,120
November	− 0.1019 (2.59)	12,777
2013		
March	− 0.1106 (3.15)	16,037
August	− 0.0877 (2.27)	13,126
2014		
February	− 0.0781 (2.00)	12,806
March	− 0.0711 (2.09)	16,092
2015		
June	− 0.0804 (2.10)	12,507
July	− 0.0777 (2.01)	12,284
2016		
March	− 0.1269 (3.13)	11,549
June	− 0.0964 (2.40)	11,522
2017		
February	− 0.0798 (2.00)	11,531
March	− 0.0982 (2.49)	11,654
April	− 0.0954 (2.67)	13,926
September	− 0.0811 (2.08)	11,992
2018		
March	− 0.0859 (2.12)	11,549
2019		
April	− 0.0835 (2.10)	10,965
September	+ 0.0776 (2.10)	13,301
2020		
March	+ 0.1229 (2.66)	9,349
April	+ 0.2173 (4.37)	7,258
May	+ 0.1589 (3.81)	9,875
June	+ 0.1594 (3.41)	7,860
October	+ 0.1351 (2.68)	6,816
November	+ 0.1296 (3.46)	12,102
December	+ 0.1666 (3.67)	8,387
2021		
January	+ 0.3464 (8.77)	11,345
February	+ 0.1594 (3.70)	9,172

Eighteen negative, 10 positive and 90 insignificantly different from zero

Controls age and its square race and country. Overall equations include year and month dummies and year equations include month dummies

It seems to be important to separate out the monthly changes in wellbeing from the regular seasonal patterns in the data, which tend to show that the early summer months of June and July have the highest wellbeing levels and January has the lowest.

Table 5 shows that the choice of month to draw a sample is crucial. We present separate results by month with our standard list of controls for year, age and its square, country of residence and race. We again present the male coefficient, t-values and sample size, overall and separately by month, for the UK, using the APS surveys for each of the three variables. We start with happiness, where four months (January, September, October and December) are significantly positive, seven are insignificantly different from zero while only one, March, is significantly negative. In the case of life satisfaction three are significantly negative (January, February and March) while nine are insignificant. For anxiety all coefficients are significantly negative with the largest negative for December and the smallest for March.

**Table 5:** Monthly regressions for wellbeing in UK = male coefficients, 2012–2021

	Happiness		Life satisfaction		Anxiety	
		N		N		N
All	+ 0.0058 (1.62)	14,53,690	− 0.0108 (3.56)	14,54,283	− 0.3746 (78.26)	14,52,481
1. January	+ 0.0351 (2.89)	1,26,738	− 0.0059 (0.58)	1,26,816	− 0.4073 (25.17)	1,26,610
2. February	0.0008 (0.06)	1,18,906	− 0.0271 (2.55)	1,18,916	− 0.3392 (20.32)	1,18,779
3. March	− 0.0381 (3.12)	1,27,540	− 0.0460 (4.45)	1,27,607	− 0.3194 (19.73)	1,27,447
4. April	− 0.0073 (0.58)	1,19,606	− 0.0152 (1.43)	1,19,095	− 0.3460 (20.78)	1,19,518
5. May	0.0051 (0.41)	1,22,249	− 0.0012 (0.11)	1,21,669	− 0.3762 (22.67)	1,22,149
6. June	− 0.0042 (0.34)	1,21,553	− 0.0142 (1.38)	1,21,607	− 0.3814 (23.08)	1,21,444
7. July	− 0.0104 (0.87)	1,24,743	− 0.0109 (1.06)	1,24,788	− 0.3341 (20.61)	1,25,231
8. August	− 0.0096 (0.80)	1,25,906	− 0.0199 (1.94)	1,25,957	− 0.3665 (22.61)	1,25,810
9. September	+ 0.0312 (2.54)	1,25,517	− 0.0136 (1.31)	1,25,593	− 0.4208 (25.75)	1,25,423
10. October	+ 0.0310 (2.35)	1,07,551	.0127 (1.15)	1,07,694	− 0.3720 (21.09)	1,07,565
11. November	0.0150 (1.19)	1,17,373	.0024 (0.22)	1,16,882	− 0.4124 (24.47)	1,17,285
12. December	+ 0.0282 (2.21)	1,14,746	+ 0.0175 (1.63)	1,14,268	− 0.4267 (25.24)	1,14,655
13. Feb-August	− 0.0096 (2.06)	8,61,659	− 0.0195 (4.95)	8,61,952	− 0.3514 (56.55)	8,60,943
14. Sept-January	+ 0.0281 (4.99)	5,92,031	0.0022 (0.47)	5,92,331	− 0.4089 (54.49)	5,91,538

Controls include country of residence, year and race. Source: APS 2012–2021. Row 13 combines February, March, April, May, June, July, August and row 14 combines September, October, November, December and January

The final two rows of Table 5 are especially remarkable. We simply split the sample by month – February through August and September through January. We include country of residence, year, month and race as controls. For the period February to August the male coefficient is negative and statistically significant (− 0.0096, t = 2.06). However, using the same model the male coefficient is positive and statistically significant for the winter months (+ 0.0281, t = 4.99). For life satisfaction the male coefficient is, again, negative and significant between February and August, but turns positive and non-significant between September and January. For anxiety, on the other hand, the male coefficient is negative and highly statistically significant in both periods of the year. Choice of months and whether the variable relates to recent events ('yesterday') thus likely drives the finding of a female happiness paradox, which can easily be driven away.

### Life satisfaction and bad mental health days in the United States

Table 6 presents results from the BRFSS on happiness and the number of bad mental health days, which also has full coverage by month. In the 4-step life satisfaction equation for the pooled years the male coefficient is significant and *positive* with a *t*-statistic of 4.01. Of the twelve, monthly life satisfaction equations, in four the male coefficient is significant and positive with a *t*-statistic over two – February, August, October and November. In December, the male coefficient is positive with a *t*-statistic of 1.70 while the remaining seven are insignificantly different from zero. None are significantly negative. This stands in direct contrast to evidence on 3-step happiness from the General Social Survey for the period 1972–2021 and sub-periods presented byBlanchflower and Bryson (2022a),^13^ where there were no significant male effects.

**Table 6:** Monthly equations in the USA–male coefficients

	Life satisfaction		#Bad mental health days			
	2005–2018	N	1993–2018	N	2019–2021	N
All	+ 0.0033 (4.01)	24,07,294	− 1.0359 (188.74)	79,68,292	− 1.2788 (71.04)	8,03,518
1. January	+ 0.0043 (1.42)	1,81,621	− 1.0157 (50.81)	5,77,893	− 1.3033 (18.26)	51,275
2. February	+ 0.0057 (2.01)	2,01,805	− 1.0332 (54.80)	6,67,095	− 1.1005 (17.66)	64,920
3. March	+ 0.0028 (1.00)	2,10,061	− 1.0423 (50.03)	6,98,420	− 1.1419 (20.79)	78,272
4. April	+ 0.0032 (1.12)	2,03,688	− 1.0302 (53.75)	6,62,018	− 1.3943 (23.26)	69,868
5. May	− 0.0031 (1.11)	2,03,357	− 1.0182 (53.04)	6,62,903	− 1.3103 (21.70)	71,071
6. June	− 0.0009 (0.32)	2,00,505	− 0.9829 (51.66)	6,65,438	− 1.2246 (19.69)	68,884
7. July	+ 0.0011 (0.37)	1,98,626	− 0.9824 (52.37)	6,73,740	− 1.1804 (19.45)	69,395
8. August	+ 0.0087 (3.04)	2,04,083	− 0.9884 (53.61)	6,87,548	− 1.2919 (20.58)	64,956
9. September	− 0.0004 (0.13)	1,94,453	− 1.0477 (54.19)	6,46,441	− 1.3329 (20.01)	60,016
10. October	+ 0.0074 (2.58)	2,06,689	− 1.0947 (57.94)	6,85,118	− 1.2565 (19.87)	68,336
11. November	+ 0.0059 (2.05)	2,01,895	− 1.0883 (58.18)	6,86,032	− 1.4920 (24.06)	71,635
12. December	+ 0.0049 (1.70)	2,00,511	− 1.0954 (56.92)	6,55,646	− 1.3849 (21.12)	64,576

Controls include state of residence, year and race. Overall equations include month dummies. Source: BRFSS, 1993–2021

We also ran separate life satisfaction equations for men and women, with controls for age, race, state and year which also included month dummies and found there were also differences in the rankings by month by gender from lowest to highest as follows.

- Men: September, July, January, May, November, March, April, June, October, December, February, August.
- Women: January, October, November, July, April, August, March, September, February, December, June, May.

It remains unclear why the month patterns are different from those reported in the UK. It is possible that climate and sunlight play a part. It does suggest the possibility that seasonal patterns by month will vary by country and by state. These are the subject of ongoing work. Of interest also is why there is much less variation by negative affect compared to positive affect variables.

The second part of Table 6 presents similar estimates for the number of bad mental health days over the prior month, coded 0–30, for the period 1993–2018. The male coefficient is negative and highly statistically significant throughout, in both the pooled regressions and the separate month regressions. The differential grew between 1993–2018 and 2019–2021.^14^ The results are reminiscent of those for anxiety in the UK, where women express greater anxiety at all times, regardless of seasonality.

**Fig. 9:**
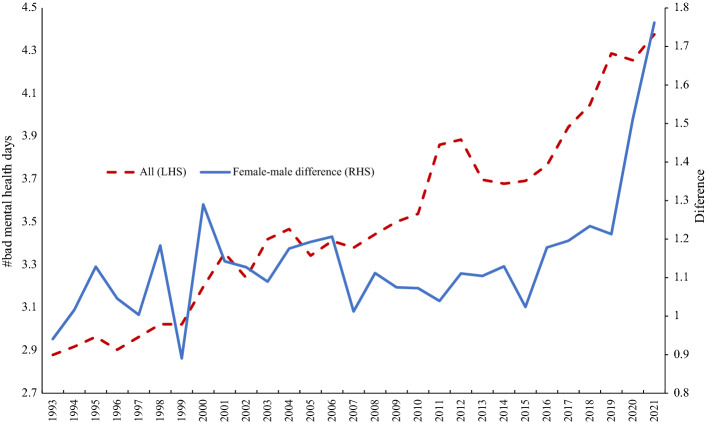
USA #Bad mental health days and female-male differential, 1993–2021 (weighted)

In Fig. 9 we plot the average number of bad mental health days, with sample weights imposed. It is clear there has been a steady rise over time. We also calculate the averages by gender and then plot the female-male difference, which is always positive, and which rises sharply from 2019 onwards. This is consistent with evidence in Blanchflower and Bryson (2022b) who find that anxiety in the USA in the period since the onset of COVID in 2020 was higher among women than men.

Table 7 reports on the lack of sensitivity of the male coefficient to changes in specification for life satisfaction and the number of bad mental health days. Column 1 does so for life satisfaction and shows that there is always a significant positive coefficient on the male variable until row i) which adds marital status to a long list of variables. The male coefficient is negative and significant when marital status is included with year, month and state dummies only (row j) or alone (row l). Analogously in the second column the same is done for the number of bad mental health days with a pooled sample for 1993–2021 with nearly nine million observations. The coefficients are always negative with coefficients around one and highly significant. We do not think it appropriate to include controls for endogenous marital status here. Men are happier than women.

**Table 7:** Sensitivity test on male coefficients after adding controls

	Male coefficient (*t* values)	
	Life satisfaction	#bad mental health days
(a) No controls	+ 0.0069 (8.26)	− 1.0503 (195.99)
(b) Add year dummies	+ 0.0064 (7.72)	− 1.0604 (197.69)
(c) Adds month dummies	+ 0.0064 (7.68)	− 1.0602 (197.64)
(d) Add state	+ 0.0054 (6.48)	− 1.0420 (194.33)
(e) Add race	+ 0.0033 (4.01)	− 1.0393 (193.87)
(f) Add age, age^2^	+ 0.0039 (4.29)	− 1.1316 (211.89)
(g) Adds labor force status	+ 0.0034 (3.70))	− 1.0445 (196.96)
(h) Adds education grade	+ 0.0024 (2.56)	− 1.0438 (196.99)
(i) Adds marital status	− 0.0237 (25.99)	− 0.9251 (172.41)
(j) Only year, month, state & marital status	− 0.0159 (19.28)	− 1.0441 (193.23)
(k) Only year, month state & labour force status	+ 0.0040 (4.34)	-0.9780 (184.00)
(l) Only marital status	− 0.0150 (18.18)	− 1.0485 (194.10)
N	24,08,878	83,98,367

Source: BRFSS. Column 1, 2005–2018, column 2, 1993–2021

## Discussion and conclusion

Across a number of studies, there is increasing evidence that men’s wellbeing has been improving relative to women’s whether that is measured using negative or positive affect variables and the gap has increased during the COVID-19 pandemic and associated lockdowns. In this paper we have shown that the trend increase in the relative wellbeing of men, captured in both happiness and life satisfaction equations, had been in train prior to COVID.

We showed how much variation by month there is in happiness and life satisfaction data. Women's wellbeing is much more volatile than men's although it seems to have greater resilience. Initially the onset of lockdown in March and April 2020 and January 2021 were greater hits to women's wellbeing than they were for men. But their wellbeing recovered faster, mostly missed by annual data. The data, however, are heavily impacted by monthly factors that we showed made a big difference especially in happiness data.

What we have seen is something quite unusual – a sign change on a variable in happiness and life satisfaction equations in the UK. In this case the male dummy goes from significantly negative to significantly positive.^15^ The question is whether this will continue in the future. Women have experienced much greater downward hits than men consistent with Becchetti and Conzo’s (2021) claim that women are relatively more affected in their satisfaction about life by good and bad events. But it does not seem they are less resilient than men or take more time to revert to their previous wellbeing levels than men do as they claim. They seem to be *more* resilient – they take a bigger hit but then seem to catch-up quickly.

We are not the first to track wellbeing during the COVID pandemic. However, those studies that have been set up to do precisely this can only examine fluctuations in wellbeing as the pandemic unfolds. They are unable to assess changes in wellbeing relative to the pre-pandemic period. This is problematic since wellbeing fell with the onset of the pandemic, as one would expect with such a health shock. This drop in wellbeing is notable in the UK, which is the focus of our investigations, but is clearly not confined to the UK. Accounting for pre-existing trends in wellbeing, and gender differences in those patterns, is potentially important given the unresolved literature on whether men’s and women’s wellbeing has been converging or diverging over time (Blanchflower and Oswald 2004 and Stevenson and Wolfers 2008, 2009). But it is also crucial because, without information on what happened to seasonal patterns in wellbeing pre-pandemic, it is difficult to know whether variance within 2020 and 2021 is part of that ‘normal’ seasonal pattern, or perhaps linked to phases of the pandemic (lockdown, school closures, new virus variants etc.).

There are also seasonal patterns in suicide which tend to peak in April-June and early summer, and they are lowest from November to January (Christodoulou et al 2012). Yu et al. (2020) examined data for 12 countries from 1986–2016 and also found that counts peaked in spring and declined in winter. Why the monthly patterns in the suicide data is the opposite of the monthly patterns in the wellbeing data remains a puzzle.

Pre-existing surveys provide an opportunity to observe what has happened to wellbeing, and differences between men’s and women’s wellbeing, pre- and post-pandemic. However, they will also struggle to distinguish between COVID effects and naturally occurring variance over time if they are unable to account for the monthly fluctuations in wellbeing, something we illustrate in this paper. Unfortunately, this is the case for a number of surveys. Most conduct survey fieldwork at a given time of year, such that survey respondents are necessarily reporting wellbeing at a given moment in the seasonal cycle. In other surveys, fieldwork timing may differ across years, or across countries, something which can profoundly affect efforts to take comparable wellbeing measures across years.

We show that gender differences and trends in wellbeing, vary markedly according to the wellbeing metric used and the frequency with which wellbeing is reported in surveys. In particular we show for the first time that there are significant variations by month in happiness data regarding whether males are happier than females, but we find little variation by month in unhappiness data. Women are consistently more anxious than men, and whilst the gap in anxiety increased during COVID, it varied little month by month. In contrast, when examining movements in positive affect one requires high frequency data throughout the year to adequately track differences in men's’ and women’s wellbeing. These monthly data reveal that women’s happiness was more adversely affected by the COVID shock than men’s, but also that women’s happiness rebounded more quickly suggesting resilience.

We find strong evidence from both happiness and unhappiness data that men are happier than women, and this is especially apparent in recent years, but there are sharp differences by month. In contrast, we find that estimates of gender differences in the subjective ill-being of men and women in the UK and the United States are insensitive to the omission of month of survey. We always find women have worse mental health than men in negative affect equations. We estimate the same happiness equation for the UK, for the pooled period 2012–2021 in the winter months of September through January and we find a positive male coefficient implying no female paradox, and one for the other months of the year that generates a negative male coefficient, implying a female paradox. Combined they generate a (weakly significant) positive effect. It seems that the female happiness paradox is a statistical illusion. Men are happier than women.

## Figures and Tables

**Fig. 10:**
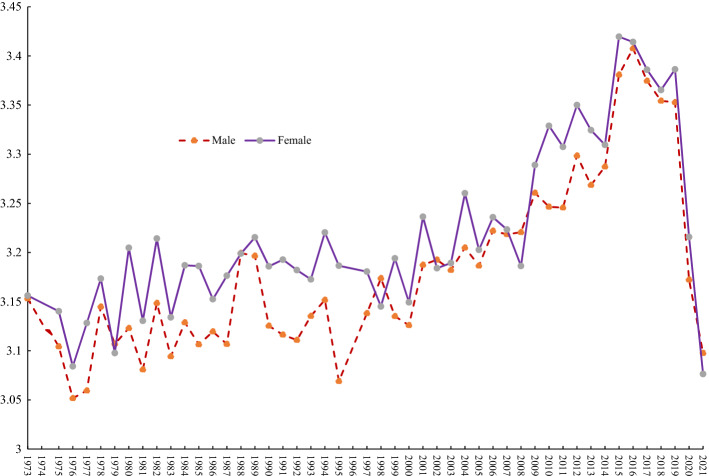
UK 4-step Life Satisfaction by Gender from Eurobarometers, 1973–2021

## Appendix

See Fig. 10 and Tables

**Table 8:** Official ONS quarterly estimates seasonally unadjusted

	Happiness	Life satisfaction	Anxious
Q2-2011	7.25	7.35	3.25
Q3-2011	7.24	7.39	3.14
Q4-2011	7.28	7.45	3.1
Q1-2012	7.33	7.43	3.06
Q2-2012	7.23	7.4	3.08
Q3-2012	7.3	7.46	3.02
Q4-2012	7.32	7.47	3.04
Q1-2013	7.34	7.46	3.03
Q2-2013	7.31	7.47	2.95
Q3-2013	7.4	7.51	2.86
Q4-2013	7.39	7.5	3
Q1-2014	7.39	7.53	2.94
Q2-2014	7.44	7.58	2.89
Q3-2014	7.45	7.58	2.87
Q4-2014	7.45	7.61	2.88
Q1-2015	7.49	7.65	2.83
Q2-2015	7.49	7.64	2.82
Q3-2015	7.44	7.64	2.84
Q4-2015	7.47	7.65	2.96
Q1-2016	7.5	7.65	2.89
Q2-2016	7.42	7.65	2.9
Q3-2016	7.51	7.66	2.87
Q4-2016	7.51	7.69	2.95
Q1-2012	7.5	7.68	2.96
Q2-2017	7.51	7.68	2.89
Q3-2017	7.51	7.69	2.9
Q4-2017	7.55	7.69	2.92
Q1-2018	7.51	7.69	2.88
Q2-2018	7.53	7.68	2.8
Q3-2018	7.54	7.7	2.87
Q4-2018	7.54	7.73	2.9
Q1-2019	7.57	7.73	2.93
Q2-2019	7.51	7.64	2.94
Q3-2019	7.51	7.64	2.92
Q4-2019	7.53	7.7	3
Q1-2020	7.4	7.67	3.26
Q2-2020	7.26	7.45	3.36
Q3-2020	7.43	7.48	3.2
Q4-2020	7.28	7.38	3.43
Q1-2021	7.31	7.33	3.23
Q2-2021	7.44	7.48	3.04

https://www.ons.gov.uk/peoplepopulationandcommunity/wellbeing/datasets/quarterlypersonalwellbeingestimatesnonseasonallyadjusted

8,

**Table 9:** Happiness, life satisfaction and anxiety by gender (weighted)

	Male	Female	Male	Female	Male	Female
2019						
September	7.51	7.47	7.62	7.64	2.67	3.21
October	7.52	7.47	7.67	7.66	2.77	3.18
November	7.51	7.47	7.7	7.71	2.77	3.16
December	7.54	7.5	7.69	7.64	2.65	3.16
2020						
January	7.43	7.33	7.61	7.57	2.75	3.33
February	7.43	7.42	7.62	7.66	2.83	3.26
March	7.09	6.93	7.52	7.49	3.64	4.28
April	7.26	7.06	7.58	7.43	3.28	3.98
May	7.44	7.37	7.57	7.51	3.02	3.61
June	7.52	7.42	7.59	7.5	2.89	3.35
July	7.54	7.6	7.6	7.54	2.88	3.19
August	7.52	7.47	7.55	7.52	2.88	3.38
September	7.48	7.44	7.45	7.44	3	3.51
October	7.36	7.25	7.34	7.29	3.11	3.67
November	7.29	7.22	7.39	7.31	3.09	3.6
December	7.29	7.13	7.42	7.23	3.13	3.68
2021						
January	7.23	6.88	7.28	7.02	3.1	3.94
February	7.31	7.22	7.34	7.15	2.99	3.47
March	7.44	7.43	7.43	7.4	2.78	3.18
April	7.58	7.54	7.53	7.51	2.72	3.22
May	7.47	7.47	7.52	7.52	2.78	3.22
June	7.58	7.55	7.55	7.51	2.73	3.17
July	7.64	7.54	7.64	7.56	2.65	3.17
August	7.57	7.52	7.61	7.55	2.81	3.31
September	7.51	7.44	7.55	7.52	2.88	3.35
October	7.26	7.33	7.45	7.45	2.96	3.53

^9^,

**Table 10:** UK happiness by month–Annual population survey

Happy	Males	Females	Male − female
12-Jan	7.25	7.26	− 0.01
12-Feb	7.24	7.28	− 0.04
12-Mar	7.31	7.42	− 0.11
12-Apr	7.22	7.29	− 0.07
12-May	7.26	7.39	− 0.13
12-Jun	7.24	7.33	− 0.09
12-Jul	7.34	7.42	− 0.08
12-Aug	7.4	7.39	0.01
12-Sep	7.29	7.32	− 0.03
12-Oct	7.28	7.28	0
12-Nov	7.2	7.3	− 0.1
12-Dec	7.32	7.38	− 0.06
13-Jan	7.18	7.22	− 0.04
13-Feb	7.34	7.31	0.03
13-Mar	7.22	7.37	− 0.15
13-Apr	7.4	7.36	0.04
13-May	7.31	7.42	− 0.11
13-Jun	7.41	7.47	− 0.06
13-Jul	7.52	7.56	− 0.04
13-Aug	7.48	7.54	− 0.06
13-Sep	7.35	7.32	0.03
13-Oct	7.33	7.34	− 0.01
13-Nov	7.32	7.36	− 0.04
13-Dec	7.37	7.42	− 0.05
14-Jan	7.25	7.3	− 0.05
14-Feb	7.29	7.39	− 0.1
14-Mar	7.42	7.5	− 0.08
14-Apr	7.45	7.5	− 0.05
14-May	7.46	7.5	− 0.04
14-Jun	7.53	7.56	− 0.03
14-Jul	7.51	7.57	− 0.06
14-Aug	7.49	7.49	0
14-Sep	7.51	7.44	0.07
14-Oct	7.4	7.38	0.02
14-Nov	7.37	7.39	− 0.02
14-Dec	7.49	7.47	0.02
15-Jan	7.38	7.42	− 0.04
15-Feb	7.5	7.46	0.04
15-Mar	7.46	7.51	− 0.05
15-Apr	7.56	7.59	− 0.03
15-May	7.53	7.57	− 0.04
15-Jun	7.52	7.59	− 0.07
15-Jul	7.46	7.55	− 0.09
15-Aug	7.5	7.53	− 0.03
15-Sep	7.49	7.42	0.07
15-Oct	7.45	7.41	0.04
15-Nov	7.38	7.41	− 0.03
15-Dec	7.49	7.48	0.01
16-Jan	7.41	7.38	0.03
16-Feb	7.43	7.41	0.02
16-Mar	7.37	7.53	− 0.16
16-Apr	7.5	7.52	− 0.02
16-May	7.55	7.6	− 0.05
16-Jun	7.47	7.53	− 0.06
16-Jul	7.56	7.58	− 0.02
16-Aug	7.62	7.66	− 0.04
16-Sep	7.51	7.53	− 0.02
16-Oct	7.47	7.46	0.01
16-Nov	7.45	7.45	0
16-Dec	7.56	7.57	− 0.01
17-Jan	7.42	7.41	0.01
17-Feb	7.44	7.52	− 0.08
17-Mar	7.43	7.55	− 0.12
17-Apr	7.57	7.64	− 0.07
17-May	7.61	7.55	0.06
17-Jun	7.57	7.58	− 0.01
17-Jul	7.62	7.61	0.01
17-Aug	7.58	7.62	− 0.04
17-Sep	7.54	7.53	0.01
17-Oct	7.5	7.54	− 0.04
17-Nov	7.53	7.52	0.01
17-Dec	7.51	7.51	0
18-Jan	7.36	7.42	− 0.06
18-Feb	7.52	7.54	− 0.02
18-Mar	7.41	7.44	− 0.03
18-Apr	7.58	7.55	0.03
18-May	7.61	7.67	− 0.06
18-Jun	7.64	7.73	− 0.09
18-Jul	7.62	7.71	− 0.09
18-Aug	7.62	7.62	0
18-Sep	7.54	7.53	0.01
18-Oct	7.58	7.49	0.09
18-Nov	7.51	7.4	0.11
18-Dec	7.56	7.55	0.01
19-Jan	7.52	7.51	0.01
19-Feb	7.53	7.54	− 0.01
19-Mar	7.53	7.56	− 0.03
19-Apr	7.65	7.65	0
19-May	7.57	7.6	− 0.03
19-Jun	7.56	7.55	0.01
19-Jul	7.6	7.62	− 0.02
19-Aug	7.58	7.59	− 0.01
19-Sep	7.51	7.47	0.04
19-Oct	7.52	7.47	0.05
19-Nov	7.51	7.47	0.04
19-Dec	7.54	7.5	0.04
20-Jan	7.43	7.33	0.1
20-Feb	7.43	7.42	0.01
20-Mar	7.09	6.93	0.16
20-Apr	7.26	7.06	0.2
20-May	7.44	7.37	0.07
20-Jun	7.52	7.42	0.1
20-Jul	7.54	7.6	− 0.06
20-Aug	7.52	7.47	0.05
20-Sep	7.48	7.44	0.04
20-Oct	7.36	7.25	0.11
20-Nov	7.29	7.22	0.07
20-Dec	7.29	7.13	0.16
21-Jan	7.23	6.88	0.35
21-Feb	7.31	7.22	0.09
21-Mar	7.44	7.43	0.01
21-Apr	7.58	7.54	0.04
21-May	7.47	7.47	0
21-Jun	7.58	7.55	0.03
21-Jul	7.64	7.54	0.1
21-Aug	7.57	7.52	0.05
21-Sep	7.51	7.44	0.07
21-Oct	7.26	7.33	− 0.07

^10^,

**Table 11:** Data by gender by survey from the ONS Opinions and Lifestyle Survey by gender for happiness and anxiety

	Happiness		Anxiety	
	Male	Female	Male	Female
20–30 March 2020	6.6	6.1	4.6	5.8
27 March–6 April 2020	6.6	6.3	4.4	5.6
3–13 April 2020	6.8	6.4	4.4	5.4
9–20 April 2020	6.9	6.9	3.9	4.4
17–27 April 2020	6.6	6.7	3.9	4.5
24 April–3 May 2020	7.1	6.6	3.6	4.5
14–17 May 2020	7.2	6.8	3.5	4.5
21–24 May 2020	7	6.8	3.9	4.2
28–31 May 2020	7.5	7.3	3.4	4
4–7 June 2020	7.1	7	3.3	4.4
11–14 June 2020	7	6.5	3.3	4.3
18–21 June 2020	7.2	6.9	3.3	4.2
25–28 June 2020	7.2	7.1	3.2	3.9
2–5 July 2020	7.1	7	3.7	4.2
8–12 July 2020	7.1	7	3.5	4.5
15–19 July 2020	7	7.2	3.7	4.2
22–26 July 2020	6.9	7.1	3.6	4.3
29 July–2 Aug 2020	7.1	7.3	3.6	4.4
5–9 Aug 2020	7.1	7.3	3.8	4.1
12–16 Aug 2020	7.3	7.1	3.4	4
26–30 Aug 2020	7.3	7.1	3.5	4.3
9–13 Sept 2020	7.2	7.2	3.7	4.3
16–20 Sept 2020	7	7	3.7	4.2
30 Sept–4 Oct 2020	7	6.8	3.8	4.8
7–11 Oct 2020	6.9	7	3.8	4.1
14–18 Oct 2020	6.9	6.9	3.9	4.7
28 Oct–1 Nov 2020	6.8	6.5	4	4.7
5–9 Nov 2020	7	6.7	3.6	4.6
11–15 Nov 2020	6.8	6.7	3.8	4.5
18–22 Nov 2020	7	6.8	3.7	4.5
25–29 Nov 2020	6.9	6.7	3.8	4.6
2–6 Dec 2020	6.9	6.8	3.7	4.5
10–13 Dec 2020	6.9	6.9	3.6	4.5
22 Dec 2020–3 Jan 2021	7	6.9	3.7	4.3
7–10 Jan 2021	6.6	6.3	4.1	5
13–17 Jan 2021	6.5	6.4	3.9	4.7
20–24 Jan 2021	6.6	6.3	3.9	4.7
27–31 Jan 2021	6.6	6.2	3.8	4.8
3–7 Feb 2021	6.7	6.3	3.7	4.6
10–14 Feb 2021	6.5	6.5	3.8	4.4
17–21 Feb 2021	6.7	6.5	3.8	4.4
24–28 Feb 2021	6.9	6.6	3.6	4.4
3–7 March 2021	6.9	6.8	3.5	4.4
10–14 March 2021	6.9	6.8	3.5	4.3
17–21 March 2021	6.9	6.8	3.5	4.4
24–28 March 2021	6.8	6.6	3.5	4.4
31 March-4 April 2021	7.1	7.2	3.4	4.1
7–11 April 2021	6.9	6.9	3.5	4.2
14–18 April 2021	7.1	7.1	3.4	4.3
21–25 April 2021	7.1	7	3.4	4.3
28 April–3 May 2021	7.1	7	3.5	4.3
5–9 May 2021	7.1	7	3.6	4.2
12–16 May 2021	7	7	3.5	4.2
19–23 May 2021	7.1	7.1	3.4	4.4
26–30 May 2021	7.3	7.3	3.3	3.9
2–6 June 2021	7.1	7.1	3.5	4.2
9–13 June 2021	7.2	7.2	3.6	4.2
16–20 June 2021	7.1	7.2	3.5	4.2
23–27 June 2021	7.1	7	3.5	4
30 June–4 July 2021	7.3	7.1	3.5	4.1
7–11 July 2021	7.1	7.1	3.7	4.3
14–18 July 2021	7.1	7.2	3.5	4.1
21–25 July 2021	7.1	7.2	3.3	4.2
28 July–1 Aug 2021	7.1	7.1	3.5	4.3
4–8 Aug 2021	7.1	7.1	3.5	4.3
11–15 Aug 2021	7.1	7.1	3.5	4.1
18–22 Aug 2021	7.2	7	3.3	4.2
27 Aug–5 Sept 2021	7.1	7.2	3.7	4.2
8–19 Sept 2021	7.1	7	3.6	4.4
22 Sept–3 Oct 2021	7.1	7.1	3.7	4.2
6–17 Oct 2021	7	7	3.7	4.2
20–31 Oct 2021	7.1	6.9	3.9	4.3
3–14 Nov 2021	7.1	7	3.5	4.2
18–28 Nov 2021	7.2	7.1	3.4	4.2
1–12 Dec 2021	7	6.9	3.4	4.4
15 Dec '21–3 Jan '22	7	7	3.9	4.4
6–16 Jan 2022	6.9	6.8	3.7	4.5
19–30 Jan 2022	7	6.8	3.6	4.1
3rd–13 Feb 2022	6.9	6.9	3.7	4.2

^11^

## Footnotes

1 See for example, Blanchflower and Clark, (2021), Blanchflower and Oswald, (2011), Fortin and Wang (2015), Stevenson and Wolfers, (2008 and 2009), Matteucci and Lima, (2016), Montgomery, (2022), Susanli (2018), Venetoklis, (2019) and Zweig (2014).

2 This observation inspired The Boomtown Rats and Bob Geldorf to compose the song “We don’t like Mondays”.

3 The authors state that they use sweeps 4–8 of the survey but these data are available in sweeps 3 and 6. They report a sample size of 63,370 which is a puzzle as there are 46,147 observations for 2006 and 53,946 for 2012. With a pooled OLS regression (n = 97,960), R^2^ = .0945 with controls for age and its square, wave, labor market status, country and years of education, the male coefficient is − 0.1491 (t = 22.36) confirming their result. In private communication Leonardo Becchetti agreed that their analysis was for 2006 only and the sample size was a typographical error.

4 A fourth question asks: "O*verall, to what extent do you feel the things you do in your life are worthwhile?*" It follows closely the path followed by life satisfaction. Since both relate to the integral of the past and we do not report separate results here.

5 Appendix Table 8 reports the official quarterly wellbeing estimates from the Office of National Statistics.

6 Appendix Table 9 reports weighted estimates for the three variables separately by gender.

7 See 'Coronavirus and the Social Impacts on Great Britain', ONS, 18 February 2022.

8 https://www.ons.gov.uk/peoplepopulationandcommunity/healthandsocialcare/healthandwellbeing/datasets/coronavirusandthesocialimpactsongreatbritaindata

9 Sample sizes on life satisfaction are as follows 2005 = 337,546; 2006 = 337,526; 2007 = 409,799; 2008 = 397,980; 2009 = 402,768; 2010 = 424,694; 2011 = 3,391; 2013 = 11,158; 2014 = 14,823; 2015 = 19,793; 2016 = 28,719; 2017 = 20,473; 2018 = 208. https://www.cdc.gov/brfss/index.html

10 To get a sense of the decline in happiness in March 2020, between 2015 and 2019 weighted APS happiness averaged 7.53. For 2020 happiness averaged 7.38 for men and 7.30 for women and 7.47 and 7.39 for women in 2021. In the case of OALS happiness averaged 7.00 for men and 6.86 for women in 2020 and 6.99 for men and 6.91 for women in 2021. The UCL Covid Social Survey has even lower happiness scores, which dropped to 5.6 for women and 6.0 for men in April 2020 and in the latest report #41 at the end of 2021 were at 6.4 for women and 6.8 for men.

11 The data are provided in Appendix Table. 11

12 Most other years in the Eurobarometer have between one and three surveys applying to very different months.

13 Blanchflower and Bryson (2022b) is an earlier discussion paper version of some of this paper.

14 These data have been examined by Blanchflower and Oswald (2017) and Blanchflower and Feir (2022) who modelled the proportion of people who said that all thirty days were bad mental health days.

15 Analogously, Blanchflower and Clark (2021) found the same in relation to the sign on children in a happiness equation. For school-aged children the sign flipped from negative to positive once controls were included for difficulty in paying the bills.
